# Building low-cost simulators for invasive ultrasound-guided procedures using the V-model

**DOI:** 10.1186/s41077-023-00254-3

**Published:** 2023-05-16

**Authors:** Vilma Johnsson, Martin Grønnebæk Tolsgaard, Olav Bennike Bjørn Petersen, Morten Bo Søndergaard Svendsen

**Affiliations:** 1 Center for Fetal Medicine, Department of Obstetrics, Copenhagen University Hospital, Rigshospitalet, Copenhagen, Denmark; 2 Department of Health and Medical Sciences, University of Copenhagen, Copenhagen, Denmark; 3 Copenhagen Academy for Medical Education and Simulation, the Capital Region of Denmark, Copenhagen, Denmark; 4 Department of Computer Science, University of Copenhagen, Copenhagen, Denmark

**Keywords:** Chorionic villus sampling, Simulator design, V-model, Ultrasound skills

## Abstract

The use of medical simulators for training technical and diagnostic skills has rapidly increased over the past decade. Yet, most available medical simulators have not been developed based on a structured evaluation of their intended uses but rather out of expected commercial value. Moreover, educators often struggle to access simulators because of cost or because no simulators have been developed for a particular procedure. In this report, we introduce “the V-model” as a conceptual framework to illustrate how simulator development can be guided by the intended uses in an iterative fashion. Applying a needs-based conceptual framework when developing simulators is important to increase the accessibility and sustainability of simulation-based medical education. It will minimize the developmental barriers and costs, while at the same time improving educational outcomes. Two new simulators for invasive ultrasound-guided procedures are used as examples, the chorionic villus sampling model and the ultrasound-guided aspiration trainer. Our conceptual framework and the use cases can serve as a template for future simulator development and documentation hereof.

## Introduction

Simulation-based medical education has revolutionized procedural skills training over the past decades [1]. There are different simulators available ranging from advanced commercial models to simpler do-it-yourself (DIY) models. It can be difficult for educators to identify relevant features of a simulator. Commercially available simulators do often impress with their physical fidelity. Yet, there is limited evidence of any association between simulator fidelity and educational outcomes [2]. Fidelity is believed to be of secondary importance relative to functional task alignment [3]. That is, if the intended use of the simulator is to support motor skills learning for novice trainees, surface characteristics are of minor importance to learning outcomes. In contrast, when the intended use of the simulator is to be used for performance assessments of a specific procedure, surface and technical similarities might influence how clinicians interact with the equipment and thereby how their performance is evaluated.

The V-model is a conceptual framework commonly used within software development. The purpose of the framework is to make sure each step of the development is guided by the intended uses [4]. However, the V-model, or any other development framework, has as far as we know never been used to guide and describe the development of simulators for medical education. Consequently, technical reports which describe the development of simulators for use within medical education often fail to support the choice of both physical and technical features in relation to the intended uses [5–7]. Understanding the intended uses and tradeoffs of a simulator brings valuable information when they are used in a training or assessment set-up. Hence, applying a needs-based approach when developing a simulator has the potential to increase the sustainability of simulation-based medical education by minimizing developmental barriers and costs, while at the same time improving educational outcomes [8].

In this paper, we have applied the V-model to guide and describe the development of two simulators where the prioritization of specific simulator features is aligned with the intended uses. Both simulators have the overall aim to enhance skills in performing ultrasound-guided fetal medicine procedure chorionic villus sampling (CVS), however, the intended uses differ. The first simulator, the CVS model, is intended for procedural skills training and assessment, and the second simulator, the ultrasound-guided aspiration (UGA) trainer, is intended for training hand–eye coordination. Our paper can serve as a template for future simulator development and reporting.

## Methods

The project took place at Copenhagen Academy for Medical Education (CAMES) between February 2020 and March 2021. The developer group included the principal designer (VJ), an engineer and technical educator (MS), a medical education scientist (MT), and a fetal medicine expert (OBP).

### The V-model

**Fig. 1:**
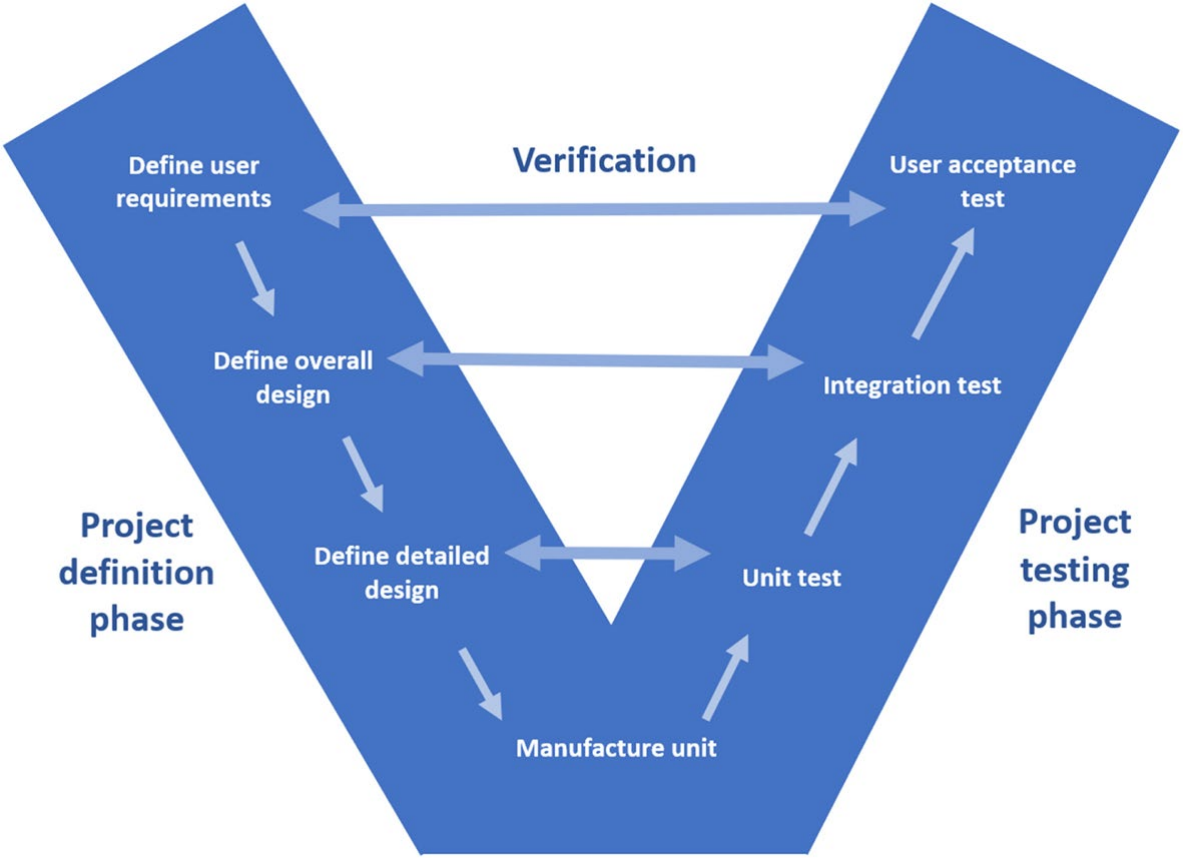
The V-model

We used a modified version of the V-model to illustrate the development of two medical simulators (Fig. 1) [4]. The V-model has two legs. The left leg is known as the project definition phase and the right leg is known as the project testing phase. The left leg starts with a user requirement analysis moving downwards to an overall design step and finally a detailed design step. Each step on the left leg has a corresponding verification step on the right leg. The right leg starts with the unit tests and moves upwards to the integration test and finally user acceptance test. The units are manufactured at the lowest level of the “V” where the two legs are connected.

The verification steps confirm that the model has been built right and corresponds to the initial requirements. All the tests of the right leg are already defined during the project definition phase. If a verification step fails, the process moves backwards. If any of the initial requirements are not feasible, the requirement document must be revised accordingly.

#### User requirements—user acceptance test

This step included an analysis by the developer group of the user needs as well as educational goals, ethical concerns, and costs [4, 8]. Interviews, literature searches, observations, and throw-away prototypes are examples of methods that were used to gather information for the requirement analysis. The defined requirements were summarized in a document that served as a guideline for the designers in the following steps. The user acceptance test included pilot testing and expert user feedback to assess how the model met the user requirements. The sample-size, user composition, and demography were determined according to the intended uses of the simulator.

#### Overall design—integration test

The designers (VJ, MS) analyzed the user requirement document and discussed possibilities and techniques by which the user requirements could be implemented. The simulators were divided into units. For each unit, physical resemblance characteristics (e.g., tactile, visual, auditory features) and functional task alignments were defined [3]. Organizing the requirements into physical resemblance characteristics and their functional task alignment helped conceptualize the design and provided a language to be used between the user and the designer [3, 8]. The integration tests were made to ensure interdependence between different units. The integration tests refer to all tests conducted where two or more units act together.

#### Detailed design—unit test

The design of each unit was specified including the selection of material. Related design files, and 3D-print files used for the models have been published under an open-source license at a public file repository, making them available for free download and usage (see “Availability of data and materials” section). Each unit was tested to verify that it corresponded to the relevant specifications in the requirement analysis. All tests performed before the units were assembled into whole or partial models are reported under the unit test, including ultrasound echogenicity if relevant.

## Results

### Chorionic villus sampling model

#### User requirements

The intended use of the CVS model was to assess skills in performing transabdominal CVS. We used the content of an assessment instrument developed in a previous international consensus study to inform user requirements [9]. Procedural steps included assessment of fetal heartbeat, selection of incision site, sampling technique, and keeping the needle in the image. The developer group identified and prioritized important anatomical features which included the skin, abdominal wall, uterus, placenta, amniotic cavity, and fetus. It was agreed that the bladder and intestines were omitted. The size of the model should correspond to a pregnant woman of about 12 weeks gestation. Physical and functional requirements are summarized in Table 1. The developers specified that reusable materials should be prioritized, and animal products should be avoided due to sanitation restrictions if the model was to be used at a hospital. Further, the developers specified that the model should be easy to assemble and take into use and replicable at low cost.

The user requirements were revised once. After several failed attempts to design a non-animal placenta that could provide a sample, we decided that a physical sample could be omitted if all the other specifications were fulfilled. Expert user feedback was used as the final verification plan for the model.

**Table 1:** Technical analysis CVS model

Unit	Physical resemblance (tactile, visual, auditory, olfactory features)	Functional task alignment
Fetus	Some resemblance of a fetus, no heartbeat necessary High echogenicity	Enhance learner engagement specifically for full procedure assessment where “check for fetal heartbeat” is an item
Amniotic cavity	Resemblance of an amniotic cavity Low echogenicity	Enhance learner engagement specifically for using safe procedural technique (i.e., avoid perforation)
Placenta	Moderate-high echogenicity Moderate-high resemblance of a placenta on ultrasound output Aspiration sample corresponding to real sample in terms of quantity	Provide a fixed target to aim that allow for needle-tracking and training or performance of safe procedural technique Context variation by changing the location (anterior/posterior/side-wall placenta)
Top layer	Nontransparent Skin surface not essential Defined ribs and hips Trackable needle with ultrasound Surface allow for many different probe-movements Acceptable surface resistance for US-probe Acceptable resistance during needle insertion Changeable thickness	To afford different probe-movements and probe-pressures to obtain the optimal view of the placenta
Base	Provide a frame around the model	Define the anatomical limits in terms of hips and ribs
Body (supplementary)	Some resemblance of hips and thorax	Provide anatomical clues and enhance learner engagement Define the upper and lower part of the model Allow for sterile dressing

#### Overall design

The design group (VJ, MS) studied commercial models and previously described DIY models. No available model fulfilled all the specified user requirements. However, ballistic gel, ultrasound gel, and silicone were found to be useful materials based on previous models. The model was divided into smaller units: the amniotic cavity and the fetus, the placenta, the top layer (Skin, Muscles, Fat, Uterus), the base, and supplementary.

#### Detailed design

**Fig. 2:**
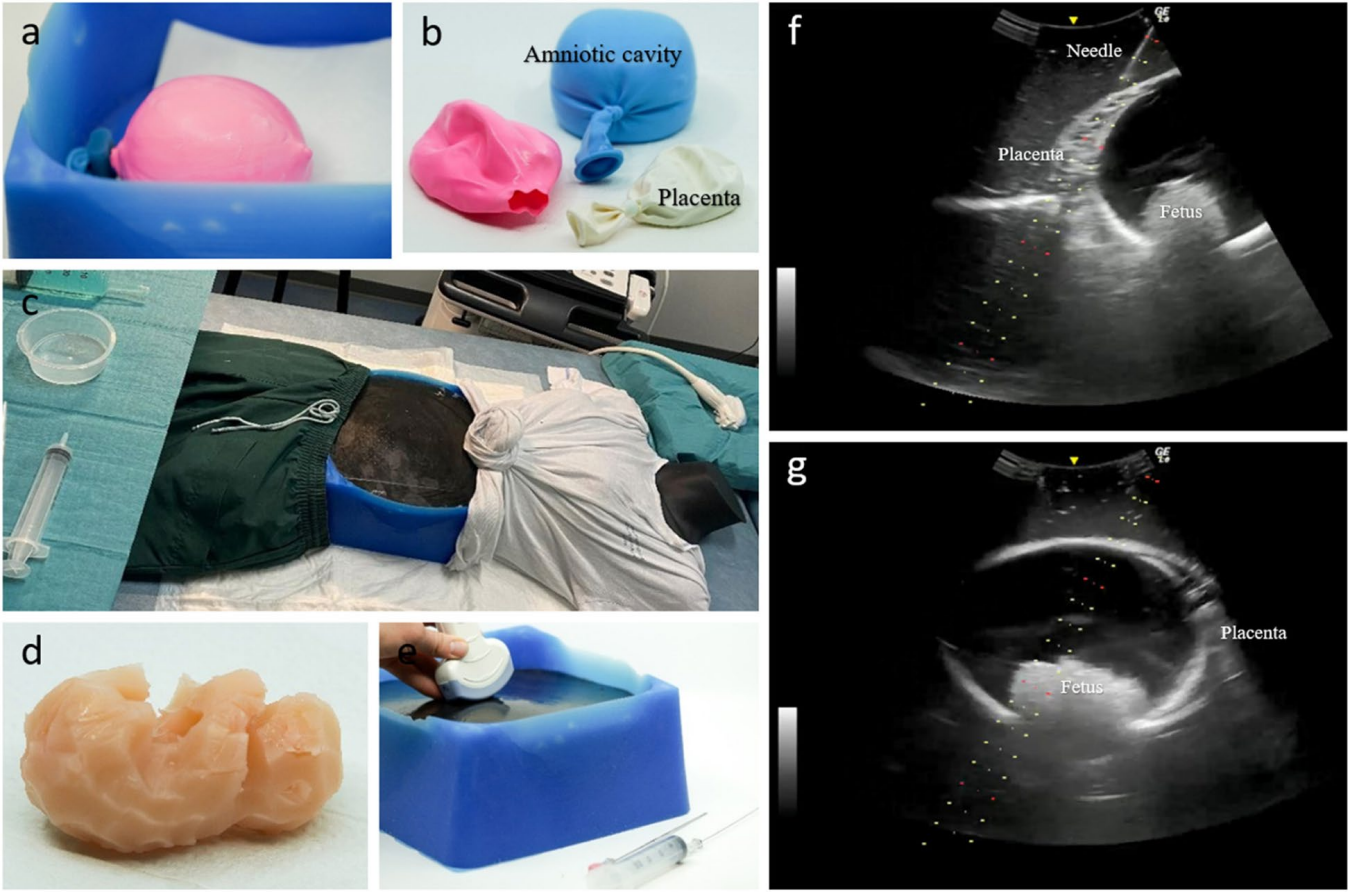
Chorionic villus sampling model. **a** Fixation of amniotic cavity compartment*. **b** Assembly balloon, amniotic cavity and placenta*. **c** Simulation set-up. **d** Fetus*. **e** CVS model*. **f**, **g** Examples of ultrasound output

Each unit of the CVS trainer was designed individually. Unit tests were performed following the requirements in Table 1 and subsequently the integration tests. Table 2 summarizes materials that were tested and their results in the unit and integration tests. The components of the final model are presented below (Fig. 2).

##### Amniotic cavity with fetus

A small fetus was carved out of a block of silicone and inserted into an 18’’ latex balloon. The balloon was filled with 280 ml water and sealed with a simple knot. The size of the balloon and volume was the result of several unit tests and integration tests where the size should correspond to the amniotic cavity of a pregnant woman of 12 weeks of gestation and afford integration with the placenta and top layer. The tests also revealed that before tying the knot, all air had to be sufflated not to interfere with the ultrasound. The size of the balloon was adaptable by changing the inserted volume of water.

##### Placenta

The placenta was made of an 18″ round latex balloon filled with 60 ml ultrasound gel. Several materials were tested and rejected in either the unit or integration test. Rejected materials included: silicone molds and condoms containing starch, toilet paper, or charcoal; tofu; and ballistic gel. The amniotic cavity and the placenta were assembled by an amputated 18″ balloon (approximately 3 cm from the inflation end). Unit testing revealed the risk of air pockets between the placenta and amniotic-cavity unit, these were eliminated by inserting a small amount of ultrasound gel between the two balloons. The placental size was adaptable by either changing the amount of inserted ultrasound gel or by using a smaller-sized balloon. The placenta could be rotated to mimic an anterior, sidewall, or posterior placenta.

##### Top layer

The top layer was made of 2.2 kg ballistic gel (Humimic Medical) mixed with 2 tablespoons graphite (Sigma-Aldrich®). The graphite enhanced the echogenicity and made the model opaque. The ballistic gel was shaped in a silicone mold. A 3D-printed plastic model created in OpenSCAD [10] was used to shape the silicone mold. The top layer was 30 cm in diameter and designed to make space for the amniotic cavity and placenta. The thickness and thinnest points were 2 cm and 7 cm respectively. The thickness of the top layer was adjustable by adding or eliminating ballistic gel. To shape the ballistic gel, it was heated in the silicone mold for 4 h in the oven at 100 °C. The graphite was carefully stirred into the hot gel when it had liquidized. The top layer was ready for use as soon as it had cooled. The top layer could be reused repeatedly. To eliminate any visible needle marks or rafts on the surface the top layer was simply reinserted in the silicone mold and heated for 2–3 h in the oven.

##### Base

A silicone base was used to assemble the amniotic cavity and placenta unit with the top layer. The silicone base also acted as a frame corresponding to the hips and ribs. A hook in the bottom of the base made sure the amniotic cavity could be fixated. A piece of moist fabric was placed at the bottom to avoid sound-wave resonance. A 3D-printed plastic model was used to design and shape the silicone base.

##### Supplementary

We supplied the model with hips and a thorax made from an old mannequin cut in half and dressed in a t-shirt and a pair of pants. The hips and thorax could be used to fixate sterile dressing if required and enhance the anatomical position of the simulator.

**Table 2:** Unit design and testing

Material *Description of materials that were tested for each unit*	Unit test *How the unit corresponds to the user requirements*	Integration test *How the unit integrates with the other units*
**Fetus**		
#1 Silicone	Low resemblance Appropriate size	Visible on ultrasound
**Amniotic cavity:**		
#1 Round latex balloon 16″ with 280 ml water	Visible on ultrasound, too small	–
#2 Round latex balloon 18″ with 280 ml water	Visible on ultrasound, appropriate size	Could be fixated easily in the base. Integrated well with the fetus and the placenta #5
#3 Round latex balloon 24″ with 280 ml water	Visible on ultrasound, too big	–
**Placenta**		
#1 Ballistic gel with red pigmentation	High resemblance, could get a sample however low resemblance to placenta Reusable	Not clearly discriminated from top layer on ultrasound
#2 Ballistic gel with graphite	Medium resemblance on ultrasound, could get a sample however low resemblance to placenta Reusable	Not clearly discriminated from top layer on ultrasound
#3 Starch with/without honeycomb in a condom	Low resemblance on ultrasound, could not provide a sample	–
#4 Starch in a silicone-mold	Silicone mold interrupted soundwaves, could not provide a sample	–
#5 Tofu	Medium resemblance on ultrasound Could get a sample however low resemblance to placenta	Clearly discriminated from top layer. Difficult to assemble with the amniotic cavity. Moderate cost
#5 Balloon with ultrasound gel	High resemblance On ultrasound Could not provide a sample	Clearly discriminated from top layer. Easily assembled with the amniotic cavity. Low cost
**Top layer**		
#1 Ballistic gel with coal + thin skin-colored silicone-layer on top	Medium/high resemblance Silicone layer would have to be remade after a certain number of uses	The silicone reduced the clarity of underlying structures on ultrasound and was difficult to fixate on the model
#2 Ballistic gel with coal	Low/medium resemblance, afford resistance during puncture, reusable	Afforded discrimination between anatomic structures below

#### User acceptance test

After approval from the developer group, one Obstetrics and Gynecology trainee and one Fetal Medicine trainee tested the model and provided feedback. They were asked to provide feedback on the degree of difficulty and their experience of using the simulator in terms of both physical and functional features. Both participants found the difficulty level appropriate. Positive reactions were provided on the visual expression of anatomical structures. However, the top layer was unstable. Consequently, the thickness of the top layer was reduced to make it more flexible and avoid the unit underneath being dislocated when probe pressure was applied. After the initial testing, eight fetal medicine experts performed two procedures on the simulator and provided their feedback. Positive reactions were provided towards the use of the simulator in an educational context and the difficulty level was rated as appropriate. Most experts commented on the viscosity of the top layer which afforded an increased resistance during the needle insertion.

### Ultrasound-guided aspiration trainer

#### User requirements

Whereas the intended use of the CVS model was to include all procedural steps of the CVS procedure, the intended use of the UGA trainer was not targeted toward a predefined anatomical location. Instead, the intention was to equip trainees with the basic technical skills needed to perform invasive ultrasound-guided procedures. More specifically, the model should afford training in hand–eye coordination, and ultrasound-guided needle incision with a focus on aiming a target while keeping the needle in the image. The simulator should include features that encouraged the use of different probe movements (tilt and rotation), aiming a target, and technical aspects of needle incision and aspiration. The target group of the UGA trainer was novices with no previous experience in ultrasound-guided procedures. As for the CVS model, reusable materials were prioritized, and the final verification test was pilot testing and expert user feedback.

#### Overall design

The user requirements were analyzed, and it was decided the model should include fixed targets that could be aimed for with a needle. Water-filled cavities were used to allow learners to practice aspiration and generate a task that could be completed. The cavities were placed in a triangular shape to make it possible to interleave the location of the cavities simply by rotating the model. A supplementary cover that would block the ultrasound waves was designed. The cover could be placed over the model with the to reduce the surface and by that encourage different probe movements.

#### Detailed design

The materials were selected based on experiences from the CVS model. The main structure “the cube” was made of 1.7 kg ballistic gel (Humimic Medical) mixed with 1 tablespoon graphite (Sigma-Aldrich®). A cubic aluminum mold commonly used for baking (PME Cake^©^) and a nodular silicone mat was used to shape the cube and its three cavities.

A prototype was used to decide what size and position the cavities should have. Nodules were carved out from a block of silicone and reshaped until they met the requirements. The distance between the surface and the target was considered as well as the size of the target since they would affect the difficulty level. Based on the prototype, the plastic model was 3D printed and used to shape the silicone mat.

**Fig. 3:**
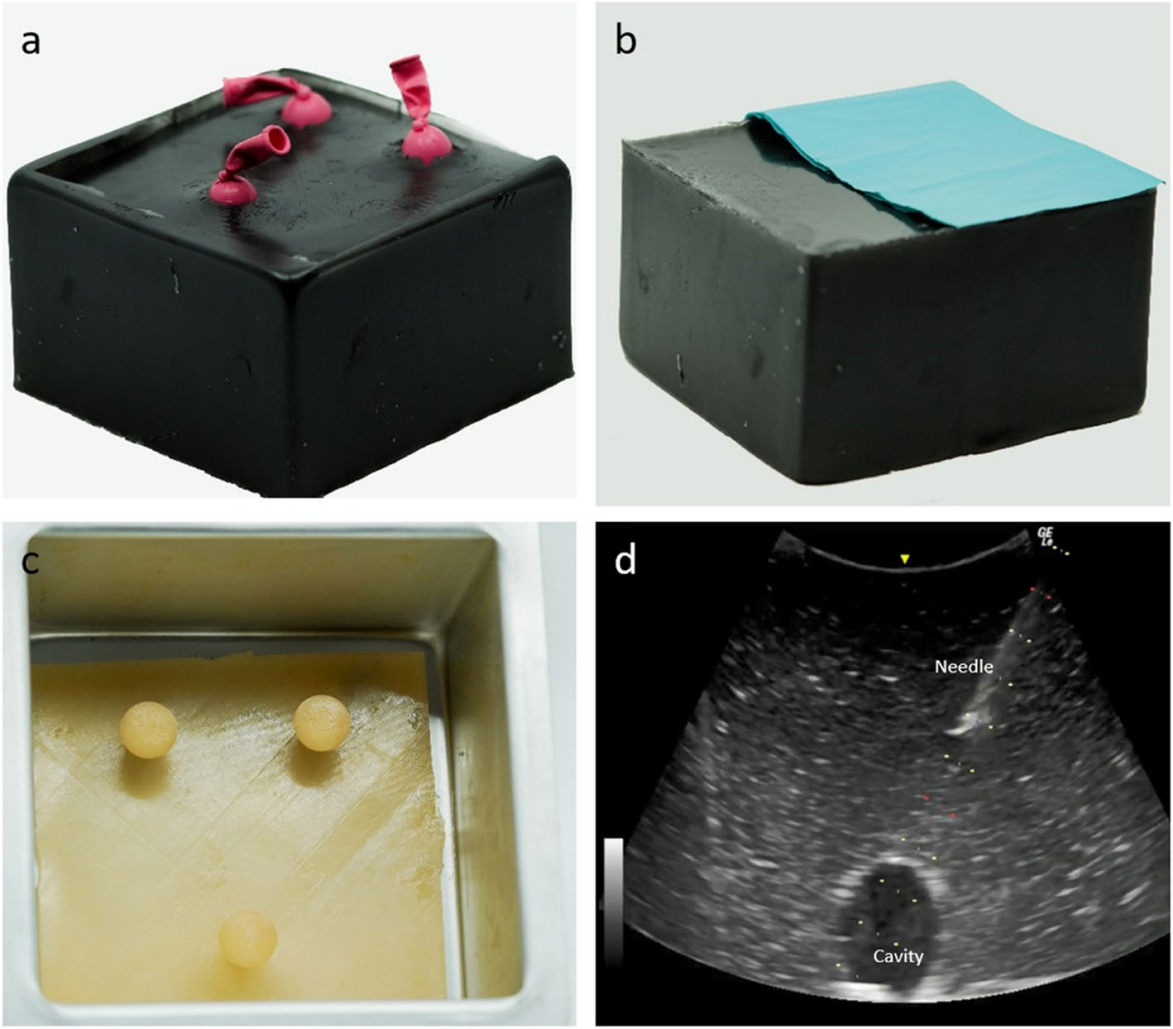
Ultrasound-guided aspiration trainer. **a** Positions of waterfilled cavities*. **b** Cover used to encourage different probe movements*. **c** Silicon-mat in aluminum mold used to shape the ballistic gel*. **d** Example of ultrasound output

One 5″ latex balloon filled with 10 ml water was placed in each cavity. A double layer of sterile dressing size 8 × 22 cm could be fixated with needles to cover half the cube (Fig. 3).

#### User acceptance test

The complete model was presented to the developer group and approved. A group of three medical students tested the model and provided feedback on the model.

### Costs

The material cost to produce one model was 321 EUR for the CVS model and 189 EUR for the UGA trainer. Total costs for the development of the models were 8910 EUR and 1710 EUR for the CVS model and UGA trainer, respectively. Working hours related to the development were far lower for the UGA trainer (1506 EUR) compared to the CVS model (7528 EUR). Costs are specified in Tables 3 and 4.

**Table 3:** Product and manufacture information

Product	Manufacturer	Quantity	Price (EUR)^a^
*CVS model*			
Amniotic cavity with fetus and placenta			
Round latex balloons 18”	Any	3/use	0.67/piece
Silicone	Dragon Skin™ FX-Pro	20 g	0.03/g
Ultrasound gel	Any	60 ml/model	n/a
60 ml syringe	Any	1	n/a
Surgical scalpel	Any	1	n/a
Top-layer and base			
Synthetic gelatin #2	Humimic Medical Gel	2.2 kg	101/kg
Silicone	Dragon Skin™ FX-Pro	~ 1200 g	0.03/g
Graphite, powder, < 20 µm, synthetic	Sigma-Aldrich®	2 tablespoons	0.07/g
3D-print	Prusament PLA	~ 1500 g	0.03/g
Body			
Plastic mannequin	Any	1	15/piece
*UGA trainer*			
Synthetic gelatin #2	Humimic Medical Gel	1.7 kg	101/kg
Round latex balloons 5”	Any	3 pieces/use	0.10/piece
Aluminum cake pan (12.7 × 12.7x10.2 cm)	PME Cake^©^	1 piece	15/piece
Silicone	Dragon Skin™ FX-Pro	100 g	0.03/g
Graphite, powder, < 20 µm, synthetic	Sigma-Aldrich®	1 tablespoon	0.07/g
3D-print	Prusament PLA	~ 260 g	0.03/g
10 ml syringe	Any	1 piece	n/a
Towel/clot	Any	1 piece	n/a
Ultrasound gel	Any	5 ml/model	n/a

^a^Prices from January 2023

**Table 4:** Cost analysis

	Cost (EUR)
**CVS model**	
Development of prototypes (100 h)	7528
Prototype materials	309
Development of 1st model (10 h)	752
Durable material	319
Consumable materials	2
*Total cost of the first model:*	8910
**UGA trainer**	
Development of prototypes (20 h)	1506
Prototype materials	15
Durable materials	189
Consumable materials	0.3
*Total cost of the first model:*	1710

## Discussion

We have applied a conceptual framework, the V-model, to guide and describe the development of two simulators for invasive ultrasound-guided procedures, the CVS model and the UGA trainer. The intended uses of the simulators guided the whole development process. The simulators were made of accessible and reusable materials with all 3D-print scripts eligible for open access.

The V-model supports that what is built corresponds to the user’s needs and introduces a common language between the user and the designer [4]. That was well illustrated by the different features of the presented simulators. The CVS model required more effort on physical resemblance characteristics that enhanced learner engagement to perform a full CVS procedure compared to the UGA trainer. Rather than decoding which organ the trainee was looking at, the focus should be on performing the procedure. Therefore, a female body shape was constructed around the simulator to invite the trainee to interact with the model and indicate an anatomical position. The UGA trainer, on the other hand, was solely intended for training in technical skills in terms of hand–eye coordination and keeping the needle in the image. Thus, the physical resemblance characteristics were irrelevant, and the main attention was directed toward the functional alignment where the model should encourage the trainee to use different techniques.

Validity frameworks such as Kane’s and Messick’s are widely used to support or refute the interpretation and uses of test scores [11, 12]. Analogous approaches are used within Medical Device Regulation (MDR) where CE marking is granted to new medical technology based on a risk classification [13]. In both cases, the intended uses and the underlying claims are essential to document and evaluate potential misuses and risks. With the emerging use of simulators within medical education, similar frameworks should be applied to simulators. Understanding how intended uses and other claims are supported by its design brings valuable information about the appropriateness to use a simulator in a learning or assessment context [14]. We used the V-model as a framework for simulator development, which allowed us to identify and test claims in a similar fashion to those used during psychometric validation and MDR. The V-model does not assess sample size or group composition, and the purpose of the study was not a full-scale end-user evaluation. Instead, the main value and contribution of applied methods lie in the use and description of the V-model for guiding the development of new simulators hoping to inspire the usage of this method in the future.

The largest expense for both simulators was the working hours used to find appropriate materials and designs. Nevertheless, using the experience from the CVS model reduced the time and effort used to design the UGA trainer. This is an important aspect of simulator development where a lot of effort are often spent on revisiting the same issues, as such “reinventing the wheel” [5, 6, 15]. A limitation of our development process was the informal format of the user acceptance test used for the CVS model and UGA trainer. Several ultrasound experts were interviewed and asked to provide feedback on the CVS model, however, not in a standardized setting. Moreover, expert reactions [16] are often insufficient as the sole source of information. Our approach, the V-model, stresses the necessity of observing user interaction and user behavior to further support the use of the CVS model and UGA trainer. As a next step, to further support the intended uses of the simulators, the collection of empirical training and assessment data on the two platforms is necessary.

## Conclusion

We have described the development of two new simulators to be used for training or assessment of invasive fetal medicine procedures using the V-model. The use of conceptual frameworks for the identification of intended uses and evaluation of underlying claims behind the development of new learning technology—including simulators—have been underprioritized until now. We suggest simulator developers apply the V-model or similar frameworks to support and document needs-based design and to increase the transparency of trade-offs made during the design phase.

## Data Availability

The 3D-scripts generated during the current study are available in the Chorionic Villus Sampling Simulator repository, https://github.com/CAMES-Engineering/Chorionic-villi-Sampling-Simulators.

## Abbreviations

CVS: Chorionic villus sampling
MDR: Medical Device Regulation
UGA: Ultrasound-guided aspiration
